# The Effect of 3D Printing Tilt Angle on the Penetration of 3D-Printed Microneedle Arrays

**DOI:** 10.3390/mi14061157

**Published:** 2023-05-30

**Authors:** Mahmood Razzaghi, Mohsen Akbari

**Affiliations:** 1 Laboratory for Innovations in Microengineering (LiME), Department of Mechanical Engineering, University of Victoria, Victoria, BC V8P 5C2, Canada; mahmoodrazzaghi@uvic.ca; 2 Terasaki Institute for Biomedical Innovations, Los Angeles, CA 90050, USA; 3 Biotechnology Center, Silesian University of Technology, Akademicka 2A, 44-100 Gliwice, Poland

**Keywords:** microneedle, microneedle array, 3D printing, penetration, tilting angle

## Abstract

Microneedle arrays (MNAs) are emerging devices that are mainly used for drug delivery and diagnostic applications through the skin. Different methods have been used to fabricate MNAs. Recently developed fabrication methods based on 3D printing have many advantages compared to conventional fabrication methods, such as faster fabrication in one step and the ability to fabricate complex structures with precise control over their geometry, form, size, and mechanical and biological properties. Despite the several advantages that 3D printing offers for the fabrication of microneedles, their poor penetration capability into the skin should be improved. MNAs need a sharp needle tip to penetrate the skin barrier layer, the stratum corneum (SC). This article presents a method to improve the penetration of 3D-printed microneedle arrays by investigating the effect of the printing angle on the penetration force of MNAs. The penetration force needed to puncture the skin for MNAs fabricated using a commercial digital light processing (DLP) printer, with different printing tilt angles (0–60°), was measured in this study. The results showed that the minimum puncture force was achieved using a 45° printing tilt angle. Using this angle, the puncture force was reduced by 38% compared to MNAs printed with a tilting angle of 0°. We also identified that a tip angle of 120° resulted in the smallest penetration force needed to puncture the skin. The outcomes of the research show that the presented method can significantly improve the penetration capability of 3D-printed MNAs into the skin.

## 1. Introduction

The skin is appealing for drug therapy because it provides an easily accessible route that does not require first-pass metabolism [1]. Microneedle arrays are comparatively new devices that could be used for drug delivery and detection through the skin. Microneedles are an excellent alternative to traditional syringe injection drug delivery systems because they allow painless penetration, minimal invasiveness, mild inflammation, and long-term treatment efficacy, and they do not need to be applied by professionals [2]. Microneedles (MNs) are primarily used for drug delivery through the skin, but they have recently been used to deliver to other tissue types, such as brain tissue [3]. In any case, the primary application of microneedle arrays involves penetrating the skin tissue. Microneedle arrays have also demonstrated great promise and some success in extracting skin interstitial fluid (ISF) for detection applications [4]. MNAs can also be used as a standalone tool when combined with microfluidic devices to develop a lab- or organ-on-a-chip. MNAs and microfluidic devices can thus be easily combined using various fabrication methods to create an advanced device for drug screening and/or organ model monitoring [5].

Fabrication strategies for MNAs include micromilling (wet and/or dry cutting) [6]; etching [7,8]; photolithography, which can also be combined with thermal- and photo-polymerization [9,10]; molding-based techniques [11,12]; cleanroom-free molding [13]; injection molding [14]; laser patterning [15]; lithography [16,17]; and photolithography with an elastocapillarity-driven self-assembly mechanism [18]. Many of these traditional fabrication methods are labor-intensive and require manual steps, limiting their cost-efficiency. As a result, MNA production requires new, accessible, and cost-effective technologies. Three-dimensional printing is a relatively new method for the production of microneedle arrays that offer numerous advantages over traditional methods. Improvements in printing resolution, feature precision, and the availability of low-cost printing raw materials have allowed the use of 3D printing to fabricate various types of MNAs [6,19,20,21]. The rapid prototyping of complex structures with precise control over their geometry, form, size, and mechanical and biological properties is possible with 3D printing [22,23,24,25]. In comparison to traditional methods, 3D printing techniques can fabricate MNA structures that are more sophisticated and complex [26]. The high resolution of recently developed 3D printers ensures the precise formation of the arrays of MNs, allowing for the manufacturing of MNAs with a variety of structures in a single step [27]. MNAs have been manufactured using different 3D printing methods, including digital light processing (DLP), high-precision stereolithography (SLA), and FDM methods [28,29,30]. Many studies have been conducted on the use of 3D-printed microneedle arrays for drug delivery [30,31,32,33,34,35,36] and diagnosis [37,38,39] applications.

Despite the fact that 3D-printed microneedles have a number of advantages over traditional MNAs, some of their specifications need to be enhanced. The poor penetration capability of 3D-printed microneedle arrays, which is brought about by the nature of the layer-by-layer production process, is one of the characteristics that needs to be improved. The physically robust stratum corneum (SC), which is the outer layer of the skin, is the layer responsible for the skin’s stiffness during the application of a microneedle array [40]. It is challenging to 3D-print a microneedle with a sharp needle tip that can penetrate the SC layer of the skin because of the layer-by-layer structure of the printed microneedle arrays. In other words, the microneedle structure cannot incorporate a sharp needle tip because it is constructed in layers during the 3D printing process, and the lower the resolution of the 3D printer, the further away from the ideal sharp shape it will be.

The height of a microneedle is recommended to be between 25 and 2000 µm [41]. The needle heights used in this work are 800 µm and 1400 µm. This is due to the fact that the microneedles should bypass the SC but not reach the nerve endings in the dermis, and pain must be absent at the time of insertion [42].

**Figure 1:**
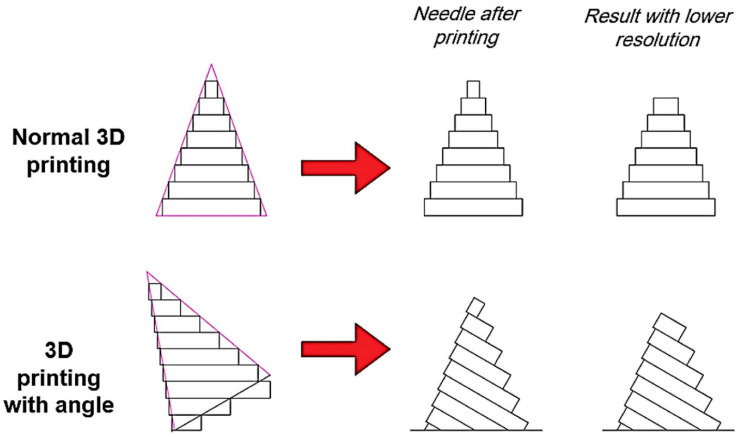
The concept of the effect of the printing tilt angle on the needle tip sharpness.

This article presents a method to improve the penetration of 3D-printed microneedle arrays by making the tip of the needle sharper to solve the abovementioned issue. The presented method is very cost-effective and easy to perform and enables us to apply sharp needles in 3D-printed microneedle arrays even when using cheap and low-resolution 3D printers. The MNAs are produced from crosslinked poly(ethylene glycol) diacrylate (PEGDA) with a molecular weight of 250 Da, which is a biocompatible hydrogel [43] and has good mechanical strength. PEGDA has been used in many research studies [26,44,45,46] as a material for 3D-printed microneedles. When compared to PEGDA materials with higher molecular weights, PEGDA, with a molecular weight of 250 Da, has relatively high stiffness and good mechanical properties, making it a good choice for MNAs, which require sufficient mechanical properties to penetrate the skin. MNAs have been 3D-printed using this type of PEGDA [26,46]. Such MNAs have been fabricated using the digital light processing (DLP) bioprinting method. This study aims to investigate the possibility of improving the penetration of 3D-printed microneedle arrays by tilting them during the printing process. In this regard, the printability of MNAs using different tilting angles is assessed and their capability for penetration is evaluated. Based on the outcomes, the MNA with the best tilting angle is suggested. The concept of using the printing tilt angle to improve the MNA’s penetration capability is depicted in Figure 1.

## 2. Materials and Methods

Materials: PEGDA (Mw 250 Da) monomer, phenylbis(2,4,6-trimethylbenzoyl)phosphine oxide (Irgacure 819), Sudan I, agarose, sodium bicarbonate, 10% neutral buffered formalin, and xylene were purchased from Sigma-Aldrich (St. Louis, MO, USA), and hematoxylin (Harris, Paul, MN, USA), acid alcohol 1%, and eosin working solution were purchased from Newcomer Supply (Middleton, WI, USA). 

Study design: This study aimed to investigate the effect of the 3D printing tilt angle on the penetration of 3D-printed microneedle arrays. In addition, the effect of the needles’ tilt angles on the penetration force was investigated. Two types of MNAs were used to perform the experiments. The penetration force of the 3D-printed MNAs with different tilt angles and needle tip angles was measured, and, based on the outcomes, the optimized condition was suggested.

Fabrication of MNAs: To prepare the PEGDA-based bioink, 2 wt% of phenylbis(2,4,6-trimethylbenzoyl)phosphine oxide as a photoinitiator and 0.04 wt% of Sudan I as a photoabsorber were dissolved in PEGDA 250. The SOLIDWORKS^®^ Version 2020 software (Dassault system, Vélizy-Villacoublay, France) was used to generate the 3D model of the MNAs and the 3D models were sliced into cross-sectional images using the CHITUBOX software (CBD-Tech, Shenzhen, China). A commercial DLP printer (Anycubic Photon Mono 4K, Shenzhen, China) was used for the 3D printing of MNAs. The layer height used for the printing was 100 µm, which can be achieved for any commercial resin 3D printer. After 3D printing, the MNAs were rinsed in ethanol for 5 min at ambient conditions to remove the unreacted bioink and then completely dried using gentle air blowing at ambient conditions. Finally, the MNAs were post-cured for 10 min using a 405-nm-wavelength UV oven to fix their shapes.

Penetration force experiment: The mechanical strength testing was performed using a custom-made compression test device. The MNAs with different printing tilt angles and needle tip angles penetrated a 3 wt% agarose hydrogel, and the changes in penetration force against displacement were measured. 

Penetration on pig skin: The MNA penetrated into pig skin after its needles were loaded with dye. The same setup as for the evaluation of the penetration force was used for the penetration of MNAs into pig skin. Before penetration, the MNA needle tips were dyed blue (with blue Tempera paint from Sargent Art Inc., Hazleton, PA, USA). The penetration performance was evaluated by visual inspection of the insertion area on the pig skin after complete microneedle penetration, and the pig skin was then washed with distilled water to prepare it for H&E staining.

To observe the area penetrated by the needle on the skin, a process of H&E staining was performed on the skin. The skin tissue was fixed with 10% neutral buffered formalin and subsequently dehydrated with increasing concentrations of ethanol, incubated in xylene, and then placed in melted paraffin wax. The tissue was then embedded in a wax block, sliced into 20 μm sections with a microtome, and floated onto glass slides. After air-drying, sections were deparaffinized with xylene, ethanol, and water. Slides were stained using the H&E staining protocol shown in Table 1. After staining, slides were incubated in ethanol and xylene, coverslipped with Entellan, and imaged with an upright microscope.

Statistical analysis: Data are presented as average values and standard deviations (SD) for the three experiments. To determine significant differences between data, the results of the test were examined using the SigmaPlot Version 12.3 software (SYSTAT Software, San Jose, CA, USA), with *p*-value < 0.05 (*), *p*-value < 0.01 (**), and *p*-value < 0.001 (***).

## 3. Results and Discussion

### 3.1. Fabrication of MNAs Using 3D Printing

**Figure 2:**
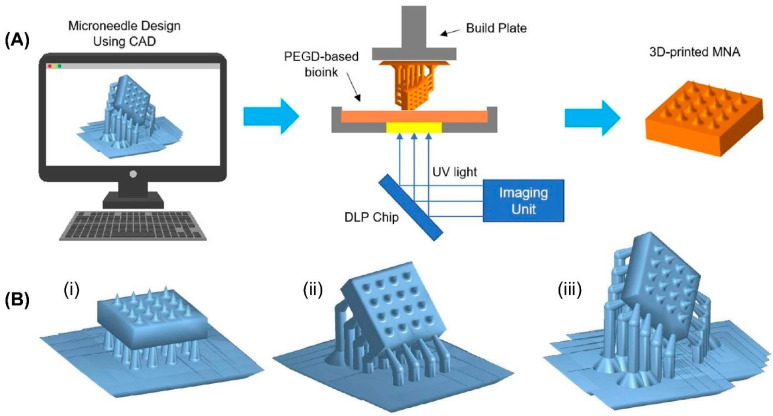
Fabrication process of microneedle arrays. (**A**) schematic illustration of DLP printing of MNAs. (**B**) Tilting of MNAs with different printing angles: (**i**) without tilting angle, (**ii**) tilting around one axis (*X* axis), (**iii**) tilting around two axes (*X* and *Y* axes).

The MNAs in this research were fabricated using the digital light processing (DLP) printing method. The process of DLP printing is shown schematically in Figure 2A. To investigate the printability of MNAs, two types of MNAs (solid and hollow) were 3D-printed. Computer-aided design (CAD) software was used to generate 3D models of both (4 by 4) solid and hollow MNAs. Digitally, the 3D model was sliced into several cross-sectional images. Every digital image was submitted to a digital micromirror device (DMD), which produced patterned light (405 nm), which was then sent through a projection lens and concentrated on the surface of a photocurable precursor solution. The projection light transformed the liquid photocurable precursor solution into a solid patterned layer. To create a 3D MNA, this process was repeated layer by layer. In this study, we used PEGDA with a molecular weight of 250 Da (PEGDA 250) as a monomer, phenylbis(2,4,6-trimethylbenzoyl)phosphine oxide (Irgacure 819) as a photoabsorber, and Sudan I as a photoinitiator to prepare the bioink.

**Figure 3:**
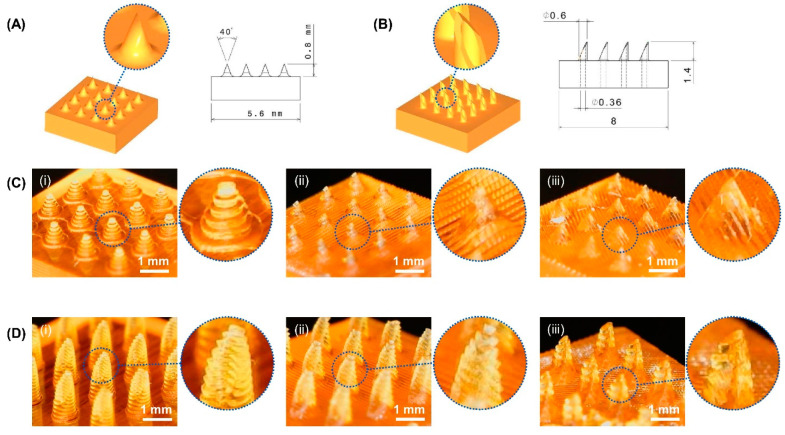
Images of 3D-printed MNAs with different printing tilt angles: (**A**) 3D model and dimensions of solid MNA; (**B**) 3D model and dimensions of hollow MNA; (**C**) 3D-printed solid MNA (**i**) without tilting angle, (**ii**) tilting 45° around one axis, (**iii**) tilting 45° around two axes; (**D**) 3D-printed hollow MNA (**i**) without tilting angle, (**ii**) tilting 45° around one axis, (**iii**) tilting 45° around two axes.

The MNAs were 3D-printed without a tilt angle, with a tilt angle around the *X* axis, and with two printing tilt angles around the *X* and *Y* axes, in order to study the effect of the printing tilt angle. These three MNA types are schematically depicted in Figure 2B. To evaluate the characteristics and conduct experiments on the samples, both solid and hollow MNAs with the dimensions and shapes shown in Figure 3A,B were designed and fabricated. Additionally, MNAs with various needle tip angles were 3D-printed in order to study the effect of the tip angle on the penetration force.

The MNAs were fabricated in two types: solid and hollow. The solid MNAs had needles with a diameter of 400 µm and a height of 800 µm. The connection point of the needle to the substrate had a corner radius of 250 µm to increase the needles’ mechanical strength. Moreover, the hollow MNAs had hollow needles with an outer diameter of 600 µm, an inner diameter of 360 µm, and a height of 1400 µm. A corner radius of 100 µm was used on the connection point of the needle to the substrate in the hollow MNAs. The needles’ tip angle for both types varied in the range of 40° to 120° (including 40°, 80°, and 120°) and the effect of the needles’ tip angle on the MNAs’ penetration force was studied. The MNAs were designed in a 4 × 4 array to make the proof of concept as simple as possible. 

Figure 3 shows images of the 3D-printed solid and hollow MNAs with different printing tilt angles. As can be seen, the 3D-printed MNAs with different tilting angles maintained their structures. 

### 3.2. Mechanical Properties

**Figure 4:**
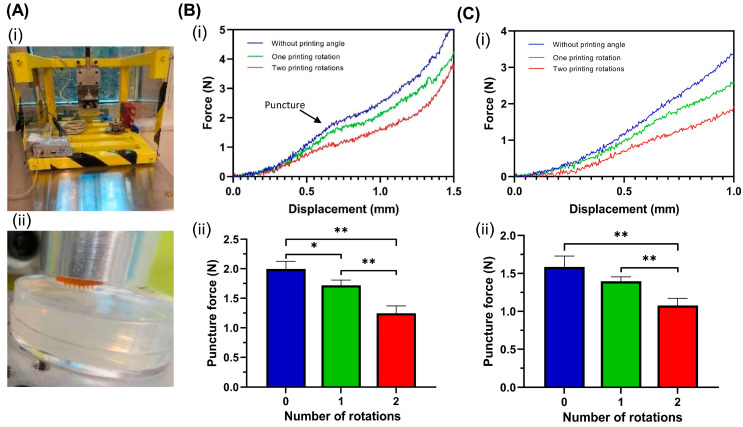
Mechanical properties of MNAs. (**A**) Testing system: (**i**) compression testing setup used to study the mechanical properties of MNAs; (**ii**) a close-up view of MNA before penetration on hydrogel. (**B**) Mechanical properties of hollow MNAs with different printing tilt angles: (**i**) changes in penetration force of hollow MNAs on hydrogel against displacement; (**ii**) puncture force of hollow MNAs 3D-printed without a printing tilt angle and with one or two tilting angles. (**C**) Mechanical properties of solid MNAs with different printing tilt angles: (**i**) changes in penetration force of solid MNAs on hydrogel against displacement; (**ii**) puncture force of solid MNAs 3D-printed without a printing tilt angle and with one or two tilting angles. (*): *p*-value < 0.05, and (**): *p*-value < 0.01.

Good mechanical properties that are sufficient to penetrate the skin are one of the main requirements when using MNAs for various applications. A compression mechanical testing system (shown in Figure 4A) was used to determine the required force for MNA penetration in a 3 wt% agarose hydrogel in order to assess the mechanical properties of the MNAs. 

To investigate the effect of the printing angle, the changes in the penetration force against displacement of the hollow and solid MNAs with a 45° tilting angle around one axis (*X* axis) and two axes (*X* and *Y* axes) were compared with the MNA without a tilting angle. As shown in Figure 4(Bi,Ci), the required force for penetration in different stages was decreased in the tilted printed MNAs compared to the MNA without a printing angle. Moreover, the required force was significantly lower in the MNAs with two rotation angles compared to those with one rotation angle. The changes in force required for penetration showed a failure to increase the force, which reflected the puncture specifications [40]. This point, which is indicated in Figure 4(Bi), is where the tip of the needle punctures the tissue. As a force is required to puncture the tissue, the penetration force is increased at a relatively rapid rate before the puncture point. The force is still increased after the puncture but at a slower rate. Figure 4(Bii,Cii) show the puncture forces for the hollow and solid MNAs without and with printing rotations. Results showed that one printing tilt angle and two printing tilt angles resulted in an approximately 14% and 38% decrease in puncture force, respectively.

**Figure 5:**
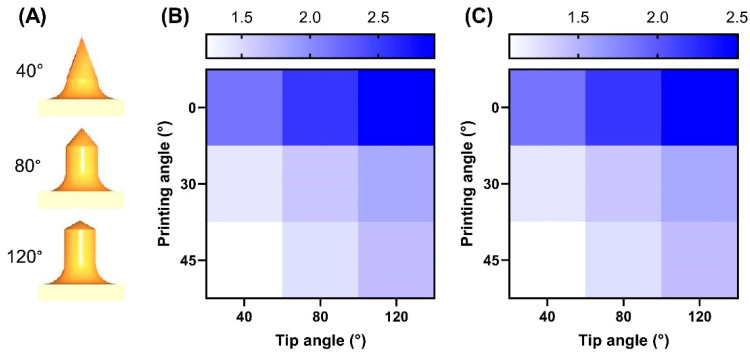
Puncture force of MNAs with different printing tilt and needle tip angles. (**A**) A schematic of needles with different tip angles used to study the effect on MNAs’ penetration force, with maps showing changes in penetration force against tip angle and printing angle for (**B**) hollow MNAs and (**C**) solid MNAs.

Another experiment was carried out to determine how the microneedle tip angle and printing tilt angle affected the force needed to puncture the MNAs. Printing tilt angles selected for this experiment were 0° (without a tilt angle), 30°, and 45°, and the tip angles were 40°, 80°, and 120°. The results of this experiment are depicted in Figure 5. Figure 5A schematically shows the different MNA tip angles used in this experiment. Figure 5B,C depict the changes in puncture force against the printing angles and MNA tip angles for hollow and solid MNAs, respectively. The results are displayed as a map because two variables affected the MNAs’ penetration force: the printing tilt angle and needle tip angle. The results showed that the penetration force decreased with the increasing printing angle but increased upon increasing the tip angle. Based on the results, an MNA with a 40° tip angle and two 45° printing tilt angles against the *X* and *Y* axes is the best MNA for penetration, with the lowest puncture force when compared to the other MNAs studied. For both hollow and solid MNAs, the same printing angle and needle tip angle were found as the best conditions.

### 3.3. Penetration of Microneedle Arrays in Pig Skin

**Figure 6:**
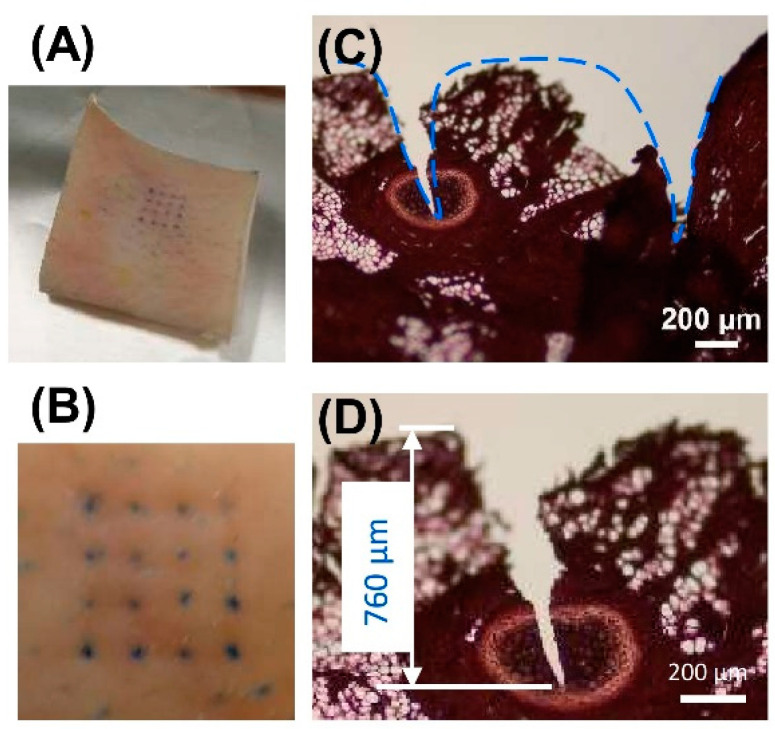
Penetration properties of MNAs. (**A**) Pig skin after penetration of dye-loaded MNA; (**B**) top view of the pig skin stained with blue dye after MNA insertion; (**C**) pig skin cross-sectional image after H&E staining, showing the penetration area on the skin; (**D**) pig skin cross-sectional image after H&E staining image of the skin, showing the penetration depth.

To evaluate the printed MNA’s performance regarding skin tissue penetration, the optimized MNA (with a 40° tip angle and two 45° printing angle rotations) was used to penetrate pig skin and the outcomes were observed. The results of this experiment are shown in Figure 6. The MNA penetrated into the pig skin after its needles were loaded with blue dye, as seen in Figure 6A,B. The pig skin was also sliced and stained after MNA penetration. The sliced and stained pig skin after MNA penetration is shown in Figure 6C. Moreover, the needle penetration depth is shown in Figure 6D. The results of this experiment demonstrated that the MNA effectively penetrated the skin and had sufficient strength.

## 4. Conclusions

In this work, we present a method to improve the penetration of 3D-printed microneedle arrays. An optimized printing tilt angle was suggested to improve the sharpness of the MNA’s tips. The results of the evaluations demonstrated that the proposed method could significantly increase the ability of the 3D-printed MNAs to penetrate the skin. The results demonstrated that the structure of the 3D-printed MNAs with a printing tilt angle could be preserved. Moreover, the outcomes showed that the use of a printing tilt angle could reduce the puncture force by 38%.

It was also realized that increasing the printing angle decreased the penetration force, while increasing the tip angle increased it. Based on the findings, an MNA with a 40° tip angle and two 45° printing tilt angles is recommended as the optimized MNA for skin penetration. Furthermore, the observations showed that the MNA effectively penetrated the skin. It can be concluded that the presented methods can be used to effectively improve the penetration performance of 3D-printed MNAs.

## Figures and Tables

**Table 1:** H&E staining protocol for deparaffinized 20 μm sections.

Reagent	Duration
Hematoxylin (Harris)	5 min
Distilled water	2 min
Differentiator (mild acid)	30 s
Distilled water	1 min
Bluing (1 g/L sodium bicarbonate)	30 s
Distilled water	1 min
95% ethanol	1 min
Eosin working solution	45 s
95% ethanol	1 min
100% ethanol	2 min (2 times)
Xylene	2 min (2 times)

## Data Availability

The data presented in this study are available on request from the corresponding author. The data are not publicly available due to privacy restrictions.

## Abbreviations

3D	Three-dimensional
CAD	Computer-aided design
DLP	Digital light processing
DMD	Digital micromirror device
FDM	Fused deposition modeling
ISF	Interstitial fluid
MN	Microneedle
MNA	Microneedle array
PEGDA	Poly(ethylene glycol) diacrylate
SC	Stratum corneum
SD	Standard deviation
SLA	Stereolithography
UV	Ultraviolet
